# Longitudinal Model-Based Biomarker Analysis of Exposure-Response Relationships in Adults with Pulmonary Tuberculosis

**DOI:** 10.1128/AAC.01794-20

**Published:** 2021-09-17

**Authors:** Andrew D. Gewitz, Belén P. Solans, William R. Mac Kenzie, Chad Heilig, William C. Whitworth, John L. Johnson, Pheona Nsubuga, Susan Dorman, Marc Weiner, Radojka M. Savic

**Affiliations:** a University of California San Francisco Schools of Pharmacy and Medicine, San Francisco, California, USA; b Centers for Disease Control and Prevention, Atlanta, Georgia, USA; c Case Western Reserve University School of Medicine and University Hospitals Cleveland Medical Center, Cleveland, Ohio, USA; d Uganda-Case Western Reserve University Research Collaboration, Kampala, Uganda; e Johns Hopkins University School of Medicine, Baltimore, Maryland, USA; f Veterans Administration Medical Center, San Antonio, Texas, USA; g University of Texas Health Science Center, San Antonio, Texas, USA

**Keywords:** mycobacterium, rifampin, time to culture conversion, treatment, biomarker, *Mycobacterium*

## Abstract

The identification of sensitive, specific, and reliable biomarkers that can be quantified in the early phases of tuberculosis treatment and predictive of long-term outcome is key for the development of an effective short-course treatment regimen. Time to positivity (TTP), a biomarker of treatment outcome against Mycobacterium tuberculosis, measures longitudinal bacterial growth in mycobacterial growth indicator tube broth culture and may be predictive of standard time to stable culture conversion (TSCC). In two randomized phase 2b trials investigating dose-ranging rifapentine (Studies 29 and 29X), 662 participants had sputum collected over 6 months where TTP, TSCC, and time to culture conversion were quantified. The goals of this *post hoc* study were to characterize longitudinal TTP profiles and to identify individual patient characteristics associated with delayed time to culture conversion. In order to do so, a nonlinear mixed-effects model describing longitudinal TTP was built. Independent variables associated with increased bacterial clearance (increased TTP), assessed by subject-specific and population-level trajectories, were higher rifapentine exposure, lower baseline grade of sputum acid-fast bacillus smear, absence of productive cough, and lower extent of lung infiltrates on radiographs. Importantly, sensitivity analysis revealed that major learning milestones in phase 2b trials, such as significant exposure-response and covariate relationships, could be detected using truncated TTP data as early as 6 weeks from start of treatment, suggesting alternative phase 2b study designs. The TTP model built depicts a novel phase 2b surrogate endpoint that can inform early assessment of experimental treatment efficacy and treatment failure or relapse in patients treated with shorter and novel TB treatment regimens, improving efficiency of phase 2 clinical trials. (The studies discussed in this paper have been registered at ClinicalTrials.gov under identifiers NCT00694629 and NCT01043575.)

## INTRODUCTION

Development of effective, safe, and well-tolerated short-course treatment regimens for drug-sensitive and resistant tuberculosis is a global health priority, but phase 3 multicenter trials to demonstrate efficacy require enormous resources and time investments (1). Sensitive, specific, and reliable biomarkers that allow the prediction of tuberculosis treatment outcomes, especially those that can be identified early, would facilitate development of new tuberculosis treatment regimens.

A rifapentine exposure-response model that used time to stable culture conversion (TSCC) in liquid cultures as a useful surrogate biomarker of treatment response in phase 2b clinical trials for tuberculosis has been described (2). TSCC describes time from the culture obtained at initiation of study treatment to the first of 2 consecutive sputum cultures negative for Mycobacterium tuberculosis that was not followed by a positive culture. The culture system used was the mycobacterial growth indicator tube (MGIT), commonly available in laboratories worldwide (3–5). TSCC biomarker analysis treated each individual time point for an MGIT culture as a binomial result (positive or negative). However, laboratory data provided by an MGIT culture are, in fact, continuous and provide the time in days from initial culture collection until the culture is identified as positive or censored at an upper quantification limit of 6 weeks (44 days) of incubation. Using a binary outcome measure of efficacy does not maximize the use of rich, longitudinal data, including serial microbiologic outcome measures as provided by MGIT culture. Therefore, modeling MGIT continuous data, otherwise termed time to positive culture or time to positivity (TTP), may be more sensitive to changes in treatment response. Time to first negative culture (or time to culture conversion [TCC]) is equivalent to the time of first censored assay value, which in practice is taken to be indicative of a negative culture readout.

In this pharmacokinetic/pharmacodynamic (PK/PD) study, TTP measurements were used to formulate an alternative model-based measure, defined by the continuous number of days on treatment at which a patient’s modeled TTP culture trajectory reached the assay censoring limit. MGIT data were obtained from two phase 2b trials for the treatment of tuberculosis (TB Trial Consortium Studies 29 and 29X).

Optimal treatment exposure can be efficiently identified with population PK/PD modeling methods that combine data on pharmacokinetic properties and efficacy outcomes. To maximize the use of available longitudinal data and identify optimal drug exposure, we used data from participants with pulmonary tuberculosis treated with rifapentine as part of a multidrug therapy and established the PK/PD relation between rifapentine exposure and time to positivity. One of the goals of modeling a longitudinal biomarker such as TTP profiles over time is to be able to identify earlier in time trial-related signals of interest, such as time to first negative culture (time to culture conversion [TCC]), compared to well-established TSCC.

Therefore, the aims of this *post hoc* study were to characterize longitudinal TTP profiles with respect to a rifapentine exposure-response relationship and to identify individual patient characteristics associated with delayed time to first negative culture, who may be unlikely to respond to shorter-term therapy. Knowledge of such phenotypes would open novel research avenues toward innovative clinical trial designs in the middle stages of drug development with the final objective of improving tuberculosis patient care.

## RESULTS

### Study population/raw data.

**FIG 1:**
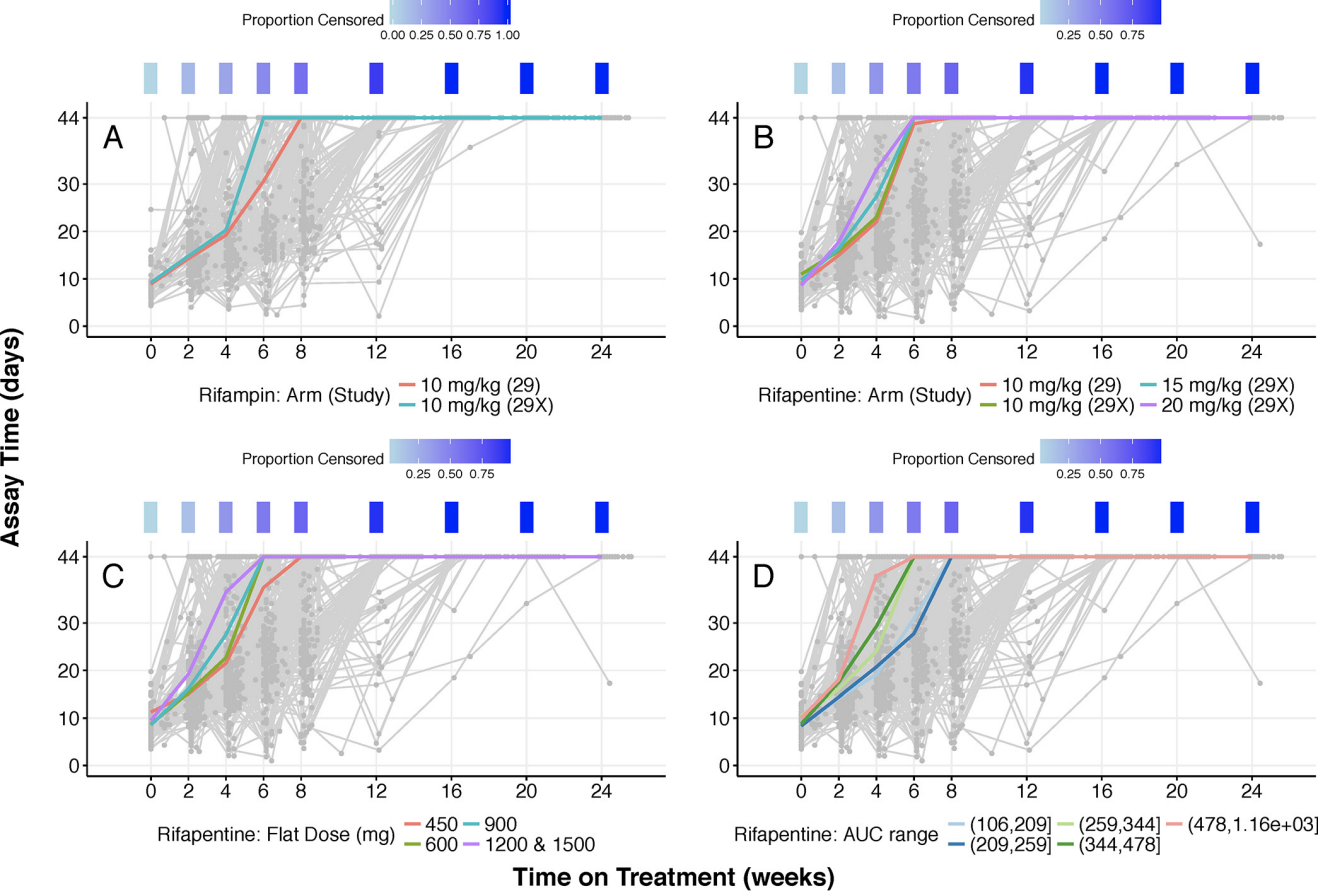
Individual trajectories of the relation between assay time (time to positivity) and time on treatment for rifampin and rifapentine. A heat map indicating the proportion of observations available at each (nominal) study time (weeks) that lies at the censoring limit (44 days) is included above each plot. (A) Rifampin raw data, grouped by dose (mg/kg). Colored lines represent median trajectories over each arm. (B) Rifapentine raw data, grouped by dose (mg/kg). Colored lines represent median trajectories over each study and arm combination. (C) Rifapentine raw data, grouped by flat dose. Colored lines represent median trajectories over each dose, as shown in the key. (D) Rifapentine raw data, grouped by quintiles of AUC_0–24_. Colored lines represent median trajectories over each steady-state AUC range, as shown in the key. Abbreviations: AUC, area under the concentration-time curve from 0 to 24 h.

Six of 668 study participants (in the modified intention-to-treat groups from TB Trial Consortium Study 29 and Study 29X) who did not have at least two MGIT results at two different (nominal) time points were excluded from the analysis. TTP pharmacodynamic analyses were performed in the remaining 662 participants, of which 408 participants received rifapentine and 254 received rifampin during intensive-phase therapy. Patient demographic characteristics can be found in Table 1. Rifapentine pharmacokinetic parameters (e.g., area under the concentration-time curve [AUC]) were obtained from a one-compartment model with first-order absorption and elimination that has been previously built and published (2), using the exposure data from Studies 29 and 29X, and were used for the rifapentine pharmacokinetic/pharmacodynamic analyses (2); note that exposure data for patients treated with rifampin were not collected in these studies, and therefore exposure to rifampin was not considered when the PK/PD model was built. Participant groups treated with rifapentine or rifampin had similar clinical and demographic characteristics at study enrollment (Table 1). Individual TTP trajectories for all patients are shown as spaghetti plots with overlaid median trajectories in Fig. 1A to D; individual panels display TTP trajectories stratified by dosing regimen and exposure. Figure S1 stratifies TTP trajectories for rifampin and rifapentine by one of the tested covariates (WHO smear grade; the categories were combined to represent high [4+, or highly bacillary, and 3+, or intermediate bacillary] and low [negative and 1+, or paucibacillary]) in order to visualize an example of covariate stratification of TTP trajectories. Raw trajectory plots showed that some subjects had TTP trajectories that reached the upper limit of assay quantification (ULOQ) in 1 measurement and later decreased below the censoring limit.

**TABLE 1:** Demographic and clinical covariates for patients who were treated for tuberculosis and had at least 2 time-to-positive-culture measurements^*a*^

Factor	Value for patients in group^*b*^		
	Rifampin (*n* = 254 [38%])	Rifapentine (*n* = 408 [62%])	Combined (*n* = 662)
Demographic factors			
Age (y)	32.9 (18.2, 77.9)	31.0 (18.1, 87.7)	31.8 (18.1, 87.7)
Race			
Black	149 (59)	245 (60)	394 (60)
White	64 (25)	77 (19)	141 (21)
Asian	30 (12)	66 (16)	96 (15)
Other	1 (<1)	3 (<1)	4 (<1)
Not reported	10 (4)	17 (4)	27 (4)
Sex, male	165 (65)	289 (71)	454 (69)
African study site	136 (54)	221 (54)	357 (54)

Clinical factors			
Dose of rifapentine (mg/kg)			
10		285 (70)	285 (43)
15		66 (16)	66 (10)
20		57 (14)	57 (9)
HIV positivity	34 (13)	36 (9)	70 (11)
Wt (kg)	55.0 (40.4, 130)	55.0 (40.0, 92.1)	55.0 (40.0, 130)
Body mass index (kg/m²)	20 (14, 41)	20 (14, 36)	20 (14, 41)
Karnofsky score			
90–100	181 (71)	255 (62)	436 (66)
60–80	73 (29)	153 (37)	226 (34)
Cough before treatment			
Productive	225 (89)	367 (90)	592 (89)
Nonproductive	19 (8)	27 (7)	46 (7)
None	10 (4)	14 (3)	24 (4)
Sputum AFB smear grade			
4+ (highly bacillary)	98 (39)	152 (37)	250 (38)
3+ (intermediate bacillary)	57 (22)	96 (24)	153 (23)
1+ (paucibacillary)	85 (33)	125 (31)	210 (32)
0 (negative)	13 (5)	32 (8)	45 (7)
Chest radiograph cavitation			
None	77 (30)	128 (31)	205 (31)
Total diam < 4 cm	82 (32)	138 (34)	220 (33)
Total diam ≥ 4 cm	94 (37)	142 (35)	236 (33)
Chest radiography, extent of diseases^*c*^			
Limited (<25% of lung)	65 (26)	80 (20)	145 (22)
Moderate (25–49%)	103 (41)	183 (45)	286 (43)
Extensive (>50%)	85 (33)	144 (35)	229 (35)
Smoking, yes	109 (43)	173 (42)	282 (42)
Food			
Empty stomach	193 (76)	185 (45)	378 (57)
Nonfat/low-fat snack	16 (6)	34 (8)	50 (8)
Food with >27 g fat	43 (17)	189 (46)	232 (35)

a Abbreviations: AFB, acid-fast bacilli; WHO, World Health Organization.

b Data are reported as median (minimum, maximum) or number (percent).

c “Limited” is defined as a lesion(s) involving a total lung area less than one-quarter the area of the entire thoracic cavity as seen in the posteroanterior (PA) or anteroposterior (AP) view. “Moderate” is defined as a lesion(s) with an area greater than that of limited lesions but, even if bilateral, involving a total lung area of less than one-half the area of the entire thoracic cavity as seen on PA or AP view. “Extensive” is defined as a lesion(s) involving a total lung area equal to or more than half the area of the entire thoracic cavity as seen in the PA or AP view.

### Base model estimation.

**FIG 2:**
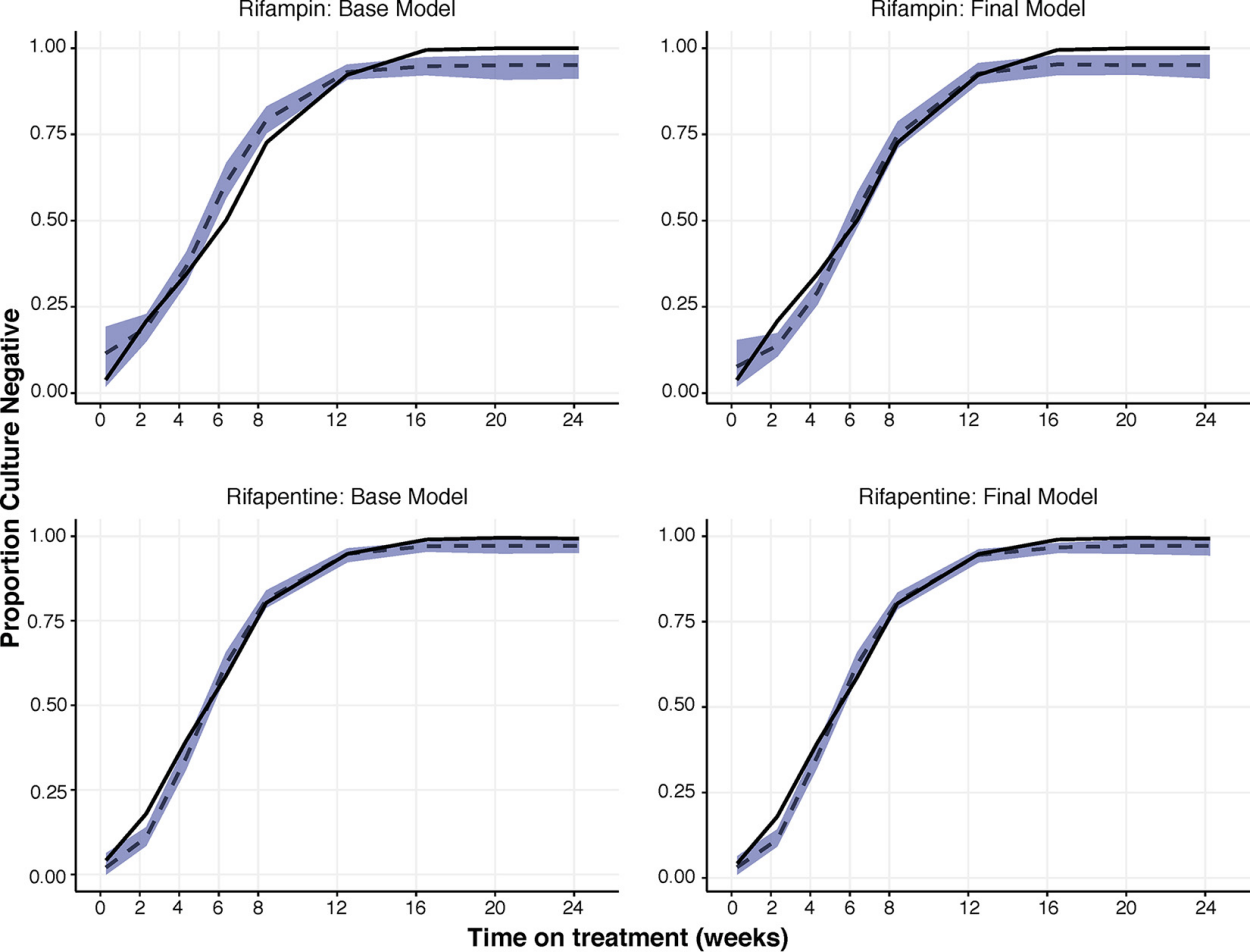
Visual predictive checks for probability of culture conversion. Base and final models of rifampin (top) and rifapentine (bottom). Observed proportion of culture-negative patients (black line), simulated proportion of culture-negative patients (dashed line), and simulated 95% confidence intervals around the simulated proportion (blue band).

Among all tested nonlinear functions, the best structural fit was provided by a shifted logistic model (see Fig. S2 in the supplemental material). This model allows for shifts in TTP trajectories due to modeled impact of pharmacokinetic or clinical covariates as visualized in Fig. S2. Estimated base and final TTP model parameters were comparable between rifampin and rifapentine treatments (Table 2; Table S1) given the choice of structural model, indicating the robustness of the shifted logistic model in capturing the dynamics of this biomarker. The most clinically meaningful relationships were quantified by the impact of covariates on the inflection point parameter (*t*_shift_) and/or slope parameter (alpha) of the TTP trajectories, illustrating faster bacterial clearance (e.g., left-shifted MGIT trajectory) and shorter time to culture conversion (e.g., time to first censored TTP value) (Fig. S1). Appropriateness of the chosen TTP model fit was supported by a visual predictive check of model-censored observations of raw and model-predicted proportions of participants, showing agreement between raw and model-predicted proportions reaching the TTP assay censoring limit of 44 days at each nominal time point (Fig. 2). It is important to mention that between days 2 and 4 for both drugs, the model slightly underpredicts the probability of having a negative culture over time, as well as after day 16 for rifampin. We attempted to address this by testing different functions (e.g., linear, exponential, 3-parameter Gompertz, and 4-parameter Weibull) and by exploring different variance-covariance structures; however, we could not improve this particular part of the curve. These underpredictions are of very small magnitude (<4%), and overall predictions are satisfactory. Further integration exercises and modeling should help create more improved model structures. The visual predictive check (VPC) shown in Fig. 2 looks at the probability of having a negative culture over time, representing the 95% confidence interval (CI) of the probability, computed using both the median trend and the interindividual variability estimated in the TTP model.

**TABLE 2:** Base and final model parameter estimates for empirical logistic models for rifampin and rifapentine, which were modeled separately^*a*^

Model	Parameter or covariate^*b*^	Rifampin		Rifapentine	
		Parameter estimate (% relative SE) or result	Interindividual variability (% relative SE)^*c*^	Parameter estimate (% relative SE) or result	Interindividual variability (% relative SE)^*c*^
Base	RUV	0.394 (2)	NE	0.388 (2)	NE
	Baseline	12.8 (6)	31.5 (9)	12.0 (2)	NE
	Time effect	0.848 (9)	36.1 (10)	0.759 (6)	45.3 (11)
	Maximum	253 (14)	NE	168 (9)	NE
	Time shift	8.53 (3)	32.7 (5)	7.69 (4)	32.6 (6)
Final	RUV	0.390 (3)	NE	0.381 (3)	NE
	Baseline	11.0 (4)	NE	12.5 (2)	NE
	Time effect	0.739 (10)	34.2 (13)	0.866 (6)	51.9 (5)
	Maximum	152 (11)	NE	162 (12)	NE
	Time shift	8.26 (5)	26.8 (6)	8.86 (5)	32.4 (7)
	Slope	NA	NA	0.301 (32)	NE

	Maximum: radiographic extent	3	0.666 (21)		
		2 (ref)	1		
		1	1.725 (61)		
	Time shift: African site			Yes (ref)	1
				No	0.866 (28)
	Maximum: smear grade	4+ or 3+ (ref)	1		
		1+ or 0	1.801 (37)		
	Time shift: smear grade			4+ or 3+ (ref)	1
				1+ or 0	0.797 (16)
	Time shift: productive cough	Productive (ref)	1	Productive (ref)	1
		Nonproductive cough	0.784 (35)	Nonproductive or no cough	0.849 (34)
		No cough	0.560 (16)		

a Abbreviations: NE, not estimated; NA, not applicable (exposure to rifampin was not available); ref, reference.

b RUV, residual unexplained variability; baseline, TTP at asymptotic baseline (prestudy); time effect, constant related to rate of change of assay time with time on treatment; maximum, maximum modeled value reachable by TTP (artifact of modeling due to censoring); time shift, treatment time to 50% of the possible maximum; slope, rate of change of exposure. For more detailed descriptions, see Fig. S1.

c Measured as percent coefficient of variation (CV).

### Rifapentine pharmacokinetic and pharmacodynamic modeling in MGIT broth cultures.

**FIG 3:**
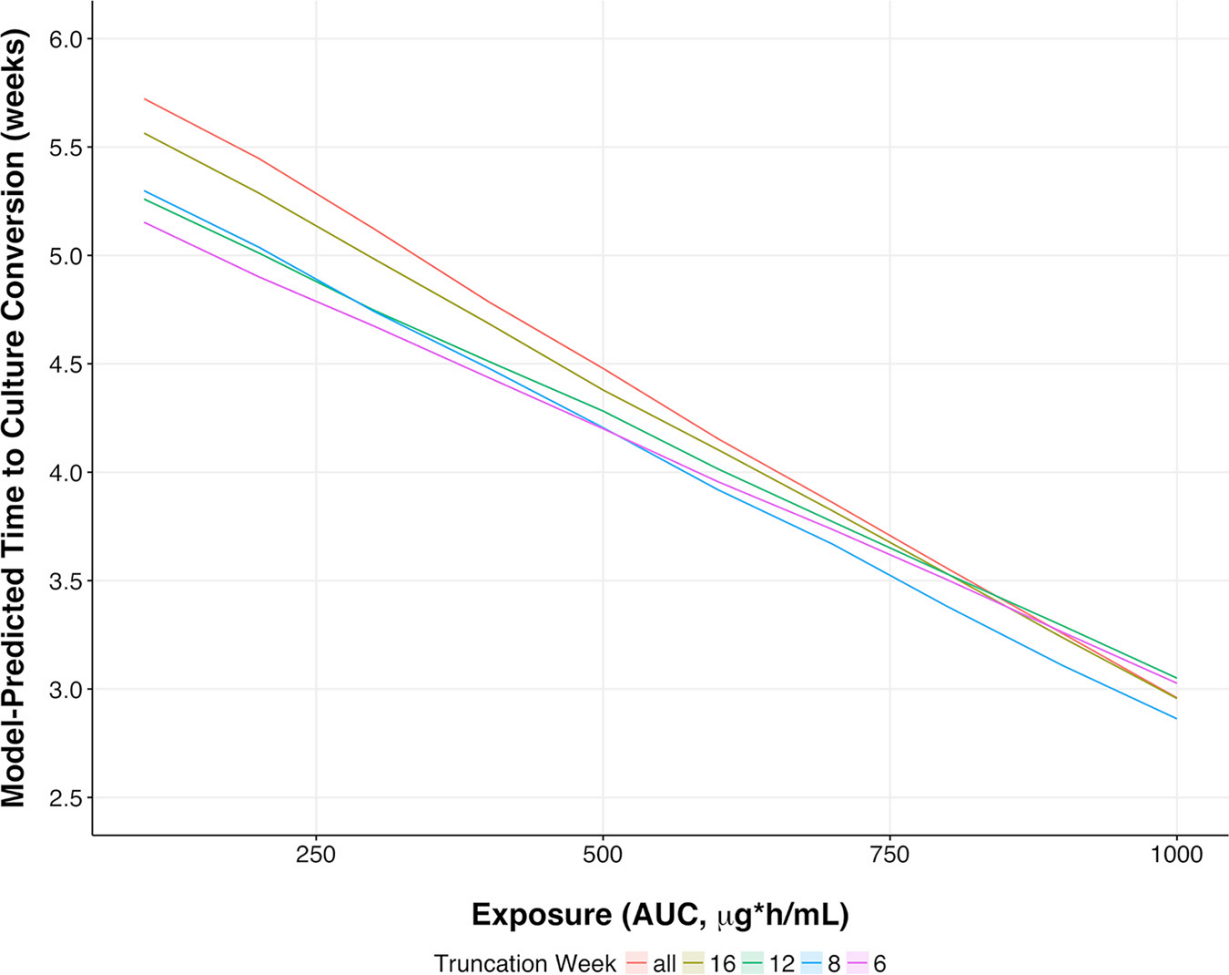
Exposure-response plots for truncated rifapentine base models. Plots show model-predicted time-to-culture conversion for logistic models fitted to the full data time course (red) and data up to 16 weeks (tan), 12 weeks (green), 8 weeks (blue), and 6 weeks (purple). Note that enough data need to be present when truncation occurs at 6, 8, or 12 weeks.

Modeling results suggested a linear relationship between either rifapentine exposure (AUC_0–24_ [AUC]) or maximum concentration (*C*_max_) and time to culture conversion in the TTP model (Fig. 3; Table S1). As mentioned, time to culture conversion is equivalent to the time of the first censored assay. These AUC-response or *C*_max_-response relations did not reach an upper limit of efficacy in the modeled data. In order to evaluate if significant exposure-response relationships can be identified with short-term data, to assess the feasibility of shorter clinical trials, individual TTP trajectories of raw data truncated at nominal study times of 6, 8, 12, and 16 weeks were evaluated. This analysis, stratifying by dosing regimen and exposure, showed that the linear relation between exposure and response was estimable and robust in trend analyses (Fig. 1 and 3). This suggests that, indeed, significant exposure-response relationships can be identified with short-term data.

**FIG 4:**
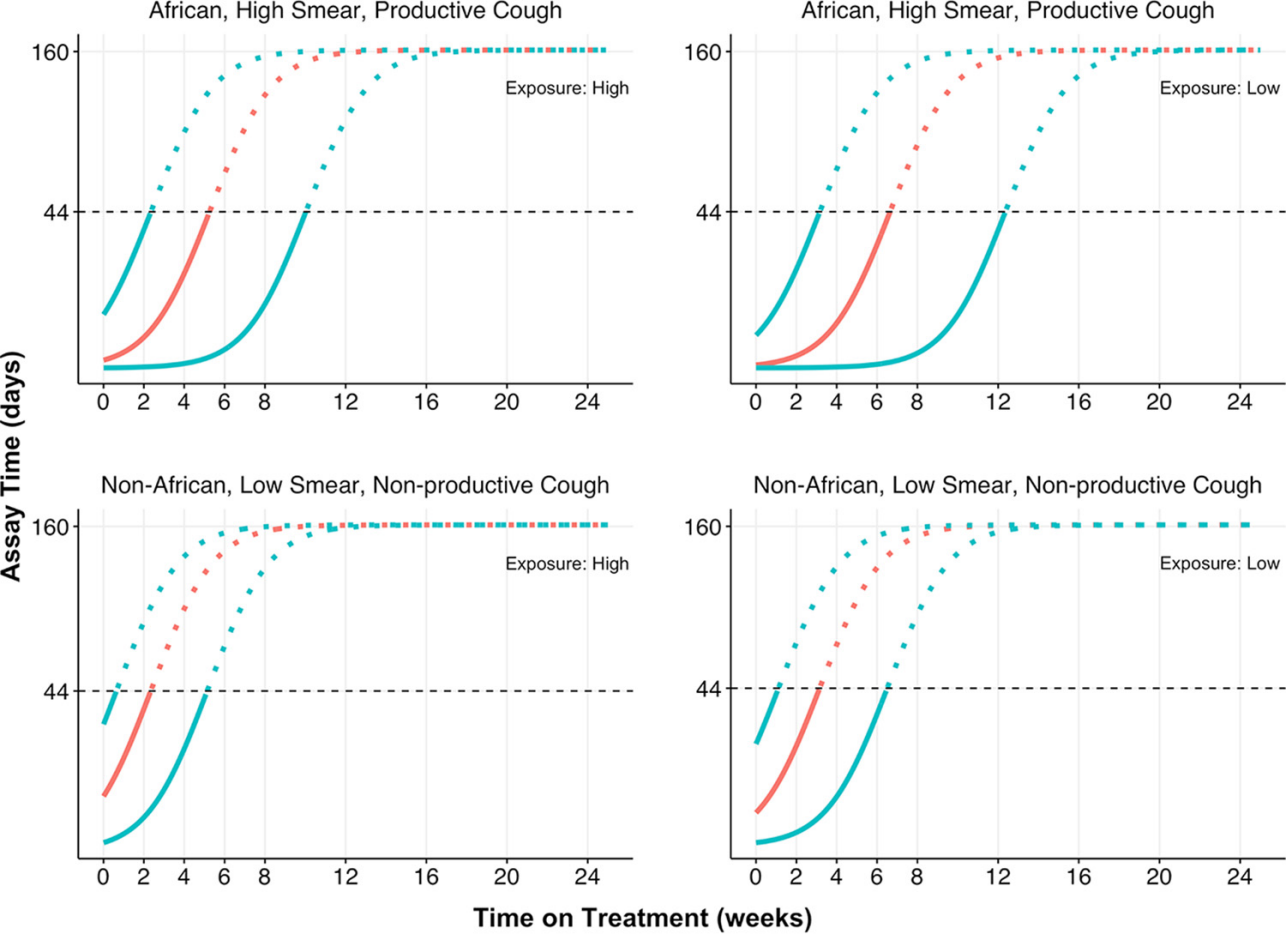
Selected model-predicted trajectories for covariate combinations for rifapentine. (Left) Covariate combinations with high exposure (AUC); (right) the same covariate combinations with low exposure (AUC). Red lines represent median simulated trajectories, and blue lines represent 90% percentiles of trajectories calculated using distribution of interindividual-variance shift in parameter only. Solid portions of trajectories indicate model-predicted trajectories corresponding to observed data (up to black horizontal dashed line at the ULOQ of 44), whereas dashed portions of trajectories represent model-predicted trajectories above the ULOQ.

In models evaluating the trajectory of TTP over time in patients treated with rifapentine, significant independent covariates included drug exposure (AUC_0–24_ or *C*_max_), sputum acid-fast bacillus (AFB) smear grade as a measure of baseline bacterial burden, baseline productive cough, and extent of disease as determined by chest radiography (Table 2). Significant independent covariates with rifampin treatment were sputum AFB smear grade, baseline productive cough, and extent of disease as determined by chest radiography (Table 2). Shorter culture conversion times were observed in participants from non-African sites, with lower burden of tuberculosis disease at baseline (as measured by WHO smear grade and cough before treatment), and with higher rifapentine exposure (Fig. 2 and 4; Fig. S3).

### Exposure-response relationships.

Exposure-response relationships for rifapentine were examined by introducing various pharmacodynamic models on each parameter of the chosen base logistic model to change model-predicted time to culture conversion. Results of these different models suggested that the most improved model fit occurred when a linear exposure-response effect of AUC was tested on the parameter controlling the offset of the independent variable of time (*t*_shift_); this confirmed the behavior suggested in the raw-data plots (Fig. 1), indicating faster bacterial clearance with higher rifapentine exposure. In the base model, the relationship between rifapentine exposure and time to culture conversion was identified as significant. Exposure affected the parameter controlling the time on treatment at which the typical population assay TTP value reached half of its modeled value, which is independent of the censoring limit. For completeness, comparable model fit (in terms of objective function value [OFV] for nested models and in terms of the Akaike information criterion [AIC] for nonnested models) was obtained using a sigmoid *E*_max_ model; OFV was significantly higher for an *E*_max_ model with the Hill coefficient (sigmoid parameter) fixed to 1. However, the sigmoid *E*_max_ model fit had a slightly larger OFV, even with 3 additional parameters. Furthermore, stable estimation of the sigmoid *E*_max_ model necessitated fixing the Hill coefficient to a large value in this model (equivalent to a step function-type effect of exposure). We selected the more parsimonious linear model to encode the effect of exposure on the shift parameter. For completeness, we tested individual *C*_max_ values as our exposure measurements and arrived at the same conclusion that a linear pharmacokinetic exposure-effect relation was the most parsimonious and best-fitting model.

There was a significant improvement in model fit when AUC was included as a linearly acting covariate on the *t*_shift_ parameter both for the full model and for models with truncated (6, 8, 12, and 16 weeks) observation sets (Table S2); similar results were obtained when *C*_max_ was used as our exposure measurement (data not shown). The exposure-response relationship for a typical patient, ranging from a model-predicted maximum of 6 weeks needed for censored TTP assay at low exposures to a minimum of 3 weeks needed for censored TTP assay over the range of areas under the concentration-time curve from 0 to 24 h (AUC_0–24_) in the base model, identified from the entire set of 3,091 longitudinal measurements, also was identified by truncating the observations after 16 weeks (2,701 observations). Comparable relationships were observed by truncating the data after 12 weeks (2,398 observations), 8 weeks (2,029 observations), and 6 weeks (1,273 observations), and quantitatively similar exposure-response relationships were derived from model fits using truncated data, confirming that models estimated using fewer and shorter nominal time points captured linear exposure-response relations over the range of calculated exposures in the population of the same effect size (Fig. 3).

### Covariate analysis.

Clinical covariates (Table 1) were tested on all logistic base models (Table S1). For each parameter, covariates that were identified as significantly affecting model fits for each drug were incorporated into the final models. Estimated covariate effects are displayed relative to a reference category containing the greatest number of patients; this coding was chosen due to the ordinal character of the selected predictors (Table 2). The marginal effects on model-predicted TCC of the category levels of each identified covariate (i.e., relative to the model with covariate effects fixed to their reference category values) was computed for rifampin; these 90% prediction intervals were computed by incorporating estimated interindividual variances on both *t*_shift_ and alpha (Table 3). For rifapentine, AUC was incorporated as a continuous covariate on *t*_shift_, and its median effect on TCC at both high (95th-percentile) and low (5th-percentile) population values on model-predicted TCC is shown, along with 90% prediction intervals; for both high and low rifapentine exposure levels, the model-predicted median TCC and associated 90% prediction intervals are likewise shown for each identified clinical covariate. Note also that the population-averaged 90% prediction intervals derived from the base models are shown (Table 3). Model-derived trajectories for selected covariate combinations for both rifampin and rifapentine showed that the chosen combinations captured the spectrum of typical TTP trajectories upon stratification by those baseline covariates identified as significant predictors of time to culture conversion. In these plots, and in order to explore the most evident change in the curves in a clear way, only interindividual variance on *t*_shift_ was included (Fig. 4). A forest plot for rifampin and rifapentine was prepared for different baseline covariate combinations, length of time (weeks) required for 95% through 99% of patients possessing those covariate groupings to achieve their first model-derived negative culture status (equivalent to the time of the first censored assay), and groupings for both rifampin and rifapentine (Fig. S3).

**TABLE 3:** Base and final model-predicted times to culture conversion^*a*^

Drug	Model	Parameter covariate	Median TCC (5%, 95%)
Rifampin	Base		6.12 (2.53, 12.25)
	Final	Reference	6.41 (3.29, 11.16)
		Radiog. extent 3	7.20 (4.15, 11.82)
		Radiog. extent 1	5.61 (2.04, 10.23)
		Smear grade 1+/0	5.50 (2.04, 10.22)
		Nonproductive cough	4.65 (2.08, 8.35)
		No cough	2.82 (0.80, 5.45)
Rifapentine	Base		5.65 (2.01, 11.26)
	Final	Reference	
		AUC_high_	5.28 (1.83, 10.30)
		AUC_low_	6.56 (2.67, 12.35)
		Non-African site	
		AUC_high_	4.33 (1.38, 8.77)
		AUC_low_	5.52 (2.09, 10.63)
		Smear grade 1+/0	
		AUC_high_	3.92 (1.12, 8.00)
		AUC_low_	4.93 (1.61, 9.83)
		Nonproductive or no cough	
		AUC_high_	4.26 (1.28, 8.46)
		AUC_low_	5.30 (1.90, 10.45)

a Abbreviations: TCC, model-predicted time to culture conversion (weeks); AUC_high_, 95th percentile of observed rifapentine 24-h AUC (689.63 μg · h/ml); AUC_high_, 5th percentile of observed rifapentine 24-h AUC (169.81 μg · h/ml). The 5 to 95% CI reported for TCC represents the influence of between-patient variability.

## DISCUSSION

Longitudinal models can incorporate time-dependent information into the evaluation of putative biomarkers (6, 7). The present study shows that a primary benefit of longitudinal biomarker modeling is the increased early identification of trial-related signals of interest compared to well-established TSCC, such as dose and exposure-response relationships, estimates of 2-month culture conversion, and hard-to-treat patient phenotypes. Models for MGIT assay TTP values have shown relationships between longitudinal TTP readout and measured bacterial load during early-phase rifampin treatment as well as more generally across various drugs and treatment regimens (8–11). TTP measurements have also been used to formulate a repeated time-to-event model in which the hazard function of interest is linked to a separate semimechanistic model for bacterial load (12).

In the present study, we modeled TTP as a censored longitudinal response variable, and our empirical models were based on the trajectories of the assay data without further assumptions about mycobacterial growth kinetics. We used this model to predict a putative surrogate (time to culture conversion), to identify an exposure-response relation for rifapentine within 6 weeks after the start of treatment, and to identify hard-to-treat patient phenotypes. For TTP, exposure-response relationship was well described by a linear relation between exposure (AUC and *C*_max_) and slope of the TTP change defining time to culture conversion. Therefore, the effect of exposure on the shortening of time to culture conversion may become more pronounced with increasing rifapentine exposure, and doses higher than those tested may help achieve higher steady-state exposures if there are no toxicity constraints. The current clinically approved dose used for rifapentine is 600 mg, and therefore, even if higher doses than those tested in these phase 2 trials are not used yet, the higher doses are candidates for improved efficacy, as recently confirmed in the phase 3 clinical trial (Tuberculosis Trials Consortium [TBTC] study 31). One of the limitations of this study is that the exposure to the active desacetyl rifapentine metabolite was not taken into account when TTP trajectories were described and therefore could be a potential point of action of model improvement. Another limitation of this study is that no measurements of free drug were available for rifapentine, and in order to establish a more accurate PK/PD relationship, protein binding would be critical. In addition, as a limitation of the present study, no rifampin exposure was available, and therefore it could not be considered.

Regarding the estimation method used in this analysis, population parameter and variability estimates were obtained using the Laplacian method. Even though this estimation method is known to use a simplified objective function due to an inconsistent estimator and provides stability issues due to second-order gradient calculations, it was selected over other estimation methods such as SAEM due to shorter computational times. To test the differences with other estimation methods, we re-estimated the final models with the Stochastic Approximation Expectation-Maximization (SAEM) algorithm. For both drugs, the parameter estimates and precision were similar between both algorithms, and so were the VPCs and clinical evaluation of the covariates. For instance, the parameter T50 with the Laplacian algorithm was estimated to be 8.86 and 8.26 weeks for rifapentine and rifampin, respectively, whereas with the SAEM method, the estimates were 8.07 and 8.11 weeks, giving differences of 9.79 and 1.82%, respectively. Taken everything into consideration, we think that the use of the Laplacian estimation method is therefore appropriate in this analysis. The present results contrast with the conclusions of our previous study in which a sigmoid *E*_max_ model best described a survival model-derived relation between rifapentine exposure and time to stable culture conversion (TSCC), with the effect of increasing exposure reaching a plateau (2). There, the results suggested that a fixed dosing scheme was sufficient to achieve this saturated response for most patients and that further increases in dose beyond 1,200 mg would offer no benefit in terms of shortening time to culture conversion while approaching limits of tolerability. However, one shortcoming of the previously published analysis includes the use of survival analysis to model a phase 2 dose selection study. This may not be ideal, because a continuous predictor (drug exposure) may be limited by issues associated with survival models, such as the identification of categorical functions of the modeled hazard rather than continuous functions (13). Another limitation of the earlier analysis is that achieving stable conversion as an outcome in the current phase 2b trial may require a minimum of 16 weeks, whereas an earlier identification of the outcome would be more useful (2).

We have identified several important patient covariates associated with slower bacterial clearance and longer time to culture conversion, such as baseline smear, productive cough, and radiographic extent of disease, all features consistent with the hard-to-treat patient phenotype (14). However, compared to the survival model from our earlier work, we were unable to detect an effect of baseline cavity size, a covariate that distinguished patients with different clinical outcomes and TSCC values (2, 4, 5). The addition of baseline cavity size did not improve the fit of the model, and therefore, keeping it as a predictor of time to positivity was not justified. Raw trajectory plots also showed that some subjects had TTP trajectories that reached the upper limit of assay quantification (ULOQ) in at least one measurement time and later decreased below the censoring limit. The nature and limitation of the chosen structural model did not allow a later decrease below the censoring limit, thereby preventing the capture of these dynamics that occurred after the response of interest was achieved. However, preliminary results from a logistic regression with response defined as the subject-specific presence or absence of a trajectory that may reach the upper limit and then fall below it showed higher odds ratios for increased levels of cavitation relative to cavitation-free chest radiograph before treatment. Alternatively, the inability of our model to capture cavitation as a predictor may have been caused by the inherent shortcomings of stepwise covariate modeling in the presence of multicollinearity between potential predictors (15).

With predictions of time to culture conversion with truncated data, any associated prediction intervals may be overly narrow, because we may not have accounted for all sources of uncertainty in our analyses, such as the effects of parametric estimation uncertainty. The quality of model predictions is also associated with aspects of study design, such as measurement timing and sample collection. Predictions from time-dependent models typically become less reliable as the horizon over which predictions are sought increases and measurements become sparser, much like for models of cross-sectional studies in which prediction intervals become wider in regions where fewer measurements have been taken (16). The present study design, with liquid culture data collected only monthly after 8 weeks, naturally led to decreased prediction accuracy of time to culture conversion for patients reaching the ULOQ after 8 weeks (when only data up to 8 weeks were used) (Fig. S2). In addition, our obtained predictions were all adjusted to the next nominal patient visit because culture status was not obtained between scheduled sputum collection visits. Therefore, there were individuals whose predictions of actual times to culture conversion using first-step model estimates were accurate (if slightly overpredicted) but for whom the subsequent rounding of the estimate to the next nominal patient-specific time decreased prediction accuracy. This problem could be mitigated with additional time points clustered around times at which most subjects who respond to treatment could be expected to achieve culture-negative status for the first time; such considerations were not a part of the original study design but could prove useful in the context of future study designs. In short, one limitation of the present study is the less frequent sample collection, especially after 8 weeks. However, frequent culture sampling is rarely feasible in the context of phase 2b clinical studies, and thus precise characterization of TSCC will be inherently limited by design and by the ability of patients to produce sputum at scheduled study visits.

Modeled TCC (as derived from measurements of assay TTP values) may be used as a surrogate for TSCC after further validation, itself routinely used as a surrogate for clinical outcomes. In most subjects, TCC may coincide with TSCC except for the trajectories of subjects who had TTP values that reached the ULOQ at 1 measurement and later decreased below the censoring limit. This (infrequent) occurrence may have been due to increased bacterial burden between these measurements or increased assay error from numerous sources at the level of low bacterial burden (2). In addition, since this analysis has been done in drug-sensitive strains, the emergence of resistance could also explain the fact that the TTP decreased below the censoring limit after reaching the ULOQ. The emergence of bacterial resistances during treatment is likely to become problematic for this mathematical model that predicts time to first culture conversion. Improved models, including more data and multidrug-resistant strains, would be needed for further evaluation and validation of the current model.

Comprehensive standardized clinical, radiographic and laboratory data collection from patients treated under directly observed therapy in two registration-quality clinical trials done in North and South America, Europe, sub-Saharan Africa, and Asia was used. The performance of MGIT cultures and drug concentrations in quality-controlled laboratories and low loss to follow-up were also reported and used. However, an additional limitation of the present modeling endeavor concerns the use of TCC as a surrogate endpoint of TSCC or long-term clinical outcome. Patients who achieve stable culture conversion later do not necessarily have poorer clinical outcomes (17), and TCC may be subject to deficiencies in surrogate quality similar to those of TSCC.

The utility of TTP trajectories in predicting clinical relapse and long-term outcomes remains undefined, both for individual probability of relapse and for trial-level relapse rates; thus, the utility of TTP as a surrogate phase 2b biomarker in tuberculosis drug development remains uncertain. Recent studies suggest that the probability of individual relapse is associated with multiple factors, including patient phenotype, adherence, and bacterial subbreakpoint MIC, as well as markers of early-treatment bacteriological response (14, 18). Regardless of the future utility of TCC as a biomarker, we have shown how longitudinal biomarker models might facilitate the identification of trial-related signals of interest at earlier times than otherwise feasible.

In addition, our modeling framework may provide useful information for drug development, with the longitudinal TTP model trajectories and parameters providing multiple potential biomarker-related characteristics that may benefit future phase 2b studies and major drug development questions at this stage, such as identification of optimal dose, identification of high-risk phenotypes and robust comparison of study arms using rich longitudinal trajectories. Further, the incorporation and modeling of individual patient characteristics that may be predictive of long-term clinical outcomes may improve the predictive efficiency of phase 2 clinical trials.

The results of this PK/PD analysis of longitudinal TTP trajectories support selection of high doses (e.g., 1,200 mg rifapentine) for late-stage clinical trials. High-risk patients, defined by high smear grade, presence of productive cough, and higher extent of lung infiltrates on radiographs, and those with high risk of suboptimal exposures might have reduced response to therapy. Our analysis on two TB drugs represents an example of how new candidate biomarkers can be used to quantify exposure-response relationship, identify important covariates, and select the dose for the next stage of clinical development. Further analysis including more example drugs and external validation of the model are warranted; however, our findings suggest that the earlier identification of dose/exposure-response relationship and impact of significant covariates is possible, opening the door for innovation of TB clinical trial designs.

## MATERIALS AND METHODS

We evaluated adults who had smear-positive pulmonary tuberculosis enrolled in 2 randomized phase 2b clinical trials that compared rifapentine with rifampin during the first 8 weeks of antituberculosis therapy (TB Trial Consortium Studies 29 and 29X) at African and non-African TB Trial Consortium sites. Detailed clinical trial design and participant characteristics in these trials were described previously (2, 16, 17). In Study 29, participants were randomized to receive rifapentine (10 mg/kg/dose) or rifampin (10 mg/kg/dose) on an empty stomach, 5 days per week for 8 weeks (intensive phase). In Study 29X, participants received rifampin (10 mg/kg/dose) or rifapentine (10, 15, or 20 mg/kg/dose) once daily with food, 7 days per week, for 8 weeks, and food consumption before pharmacokinetic sampling was documented with detailed food history. In both studies, participants were treated during the continuation phase with rifampin and isoniazid according to published guidelines (19). Methods and results of both treatment trials were published previously (20, 21). The pharmacokinetic and pharmacodynamic analysis included participants from the modified intention-to-treat group (individuals with culture-confirmed M. tuberculosis at study entry which was sensitive to isoniazid, rifampin, and pyrazinamide) from both treatment studies. The studies were approved by the Institutional Review Boards of the United States Centers for Disease Control and Prevention and participating sites. Informed consent was obtained from all participants. Both trials were registered at ClinicalTrials.gov (NCT00694629 and NCT01043575).

### Assay details.

The MGIT system, which currently is the most widely used liquid-medium sputum culture system for mycobacteria, provided a faster and more sensitive alternative to traditional culture techniques such as solid-medium Lowenstein-Jensen culture or AFB smear microscopy (18). The MGIT assay yields readouts of the time (in days) required to observe detectable fluorescence, indicating the presence of active mycobacterial oxygen consumption (time to positivity [TTP]) in sputum samples cultured in liquid medium (3). In this study, larger TTP values were representative of smaller bacterial burden in cultured samples, up to an assay time limit of 44 days.

Longitudinal TTP data were collected in these two phase 2b trials (Study 29 and Study 29X), with patient samples collected at enrollment into the trials; after completion of 2, 4, 6, and 8 weeks of treatment (during the intensive phase); and then monthly during continuation-phase treatment up to 24 weeks. All cultures obtained before trial enrollment (defined as baseline or 0 days) were excluded from the analysis. Two sputum cultures were obtained routinely after 8 weeks of therapy (at the end of intensive-phase therapy), and all available sputum culture results were included in the analyses.

### Model development. (i) Structural models.

Efficacy endpoints were characterized using serial sputum culture results from MGIT cultures. We used the time-dependent trajectory to characterize the population-average trajectory, with individual trajectories parameterized by subject-specific random effects and baseline covariates (see the supplemental material). The model-predicted time on treatment used the participant TTP MGIT tests that first reached the upper censoring limit of 44 days. Covariates tested in exposure-response models included age, sex, weight, race, HIV status, body mass index, the summed diameter of all cavitary lesions on pretreatment chest radiographs, extent of lung infiltrate, baseline sputum smear grade, presence of productive or nonproductive cough, and Karnofsky score.

Various models were tested and evaluated for fit, stability, and parsimony (Table S1). The changes in the time-dependent trajectory, when a parameter was changed while holding all others fixed, were shown in schematic diagrams of the base logistic models with a time shift in the independent variable (Fig. S1). The dependent variable *t*_assay_ was defined as MGIT assay time (days), and the independent variable/covariate *t*_treatment_ was defined as time (weeks) on treatment. The M3-based method of augmenting the likelihood of noncensored observations, with the likelihood of censored observations being greater than the censoring limit, was used because of the right-censored character of the longitudinal data (TTP values of >44 days were treated as being above the ULOQ) (22, 23).

### (ii) Exposure-response modeling.

Data on exposure to rifampin were not collected in these studies, and therefore, exposure to rifampin was not considered when the PK/PD model was built. On the other hand, the drug effect of rifapentine was evaluated as a dose (fixed [mg] and weight-based [mg/kg]) and as a steady-state AUC_0–24_ derived from the established population PK model for the population parameters of the logistic base models (linear, *E*_max_, and sigmoid *E*_max_ models).

The effect of time on treatment was entered as a covariate into the base structural models (Table S1). All other covariates (Table 1) were evaluated using stepwise covariate modeling for rifampin and rifapentine by forward selection (*P < *0.05) and backward elimination (*P < *0.01).

All nonlinear mixed-effects models were developed with pharmacokinetic modeling software (NONMEM 7.3.0; Icon Inc., Verona, PA), and other development was performed with the statistical and modeling software R and Pirana (24, 25). Population parameter and variability estimates were obtained using the Laplacian estimation method. Even though this estimation method is known to use a simplified objective function due to an inconsistent estimator and provides stability issues due to second-order gradient calculations, it was selected over other estimation methods such as SAEM due to much shorter computational times. However, all final models were rerun with the SAEM algorithm, and no major changes were found.

Model selection was carried out using a combination of objective function value (OFV), goodness-of-fit plots, and model stability analysis (model convergence). When two hierarchical models were compared, likelihood ratio tests for nested models were used to assess statistical significance. Nonnested models were compared statistically using the Akaike information criterion [AIC].
