# Supplementary material for: Longitudinal Model-Based Biomarker Analysis of Exposure-Response Relationships in Adults with Pulmonary Tuberculosis
Source: Antimicrob Agents Chemother. 2021 Sep 17;65(10):e01794-20. doi: 10.1128/AAC.01794-20 (PMC8448095; doi:10.1128/AAC.01794-20)

Supplementary Materials

SUPPLEMENTARY TABLE 1 Empirical models: structural model forms and parameterizations with descriptions*

Model	Structural Form	Properties
Linear	$t_{assay} = t_{base} + \alpha \cdot t_{treatment}$	Minimum OFV, 9950<br>Reasonable stability
Exponential	$t_{max} - (t_{max} - t_{base})exp(-\alpha \cdot t_{treatment})$	Minimum OFV, 7401<br>Reasonable stability
Shifted logistic†	$t_{assay} = t_{base} + \frac{t_{max} - t_{base}}{1 + e^{-\alpha(t_{treatment} - t_{shift})}}$<br>--<br>$t_{shift} = t_{shift,pop}(1 - \beta \cdot PK_{meas})$	Minimum OFV, 7233<br>Reasonable stability
3-parameter Gompertz	$t_{assay} = t_{max} \cdot \exp(-t_{base} \cdot e^{-\alpha \cdot t_{treatment}})$	Minimum OFV, 8743<br>Poor stability
4-parameter Weibull	$t_{assay} = t_{max} - (t_{max} - t_{base}) \cdot e^{-(\alpha \cdot t_{treatment})^{\delta}}$	Minimum OFV: 9048<br>Poor stability

Parameter	Description
t_{max} (d)	Maximum modeled value reachable by TTP (artifact of modeling due to censoring from above)
t_{base} (d)	TTP at asymptotic baseline (pre-study)
t_{shift} (wk)	Treatment time to 50% of their possible maximum
α	constant related to rate-of-change of assay time with time-on-treatment
β	rate of change of exposure/concentration (linear)
δ	controls the time-abscissa for Weibull model inflection point

†Top, chosen structural model; bottom, exposure-response model. Parameter chosen for exposure-response is colored in red. Minimum OFV shown for base model (without exposure). ***Abbreviations***: OFV, objective function value; PK_{meas} , exposure (AUC, C_{max}) or concentration; pop, estimated population value of parameter

SUPPLEMENTARY TABLE 2 Model-based significance of area under the curve from 0 to 24 h as a covariate for full and truncated base models

Rifapentine Model	dOFV	*P* ≤*
Full model (all data)	21.074	.00001
Observations		
≤ 16 wk	14.74	.0002
≤ 12 wk	12.758	.0004
≤ 8 wk	13.019	.0003
≤ 6 wk	7.219	.008

Abbreviation: $AUC_{0\text{-}24}$, area under the concentration-time curve from 0 to 24 h; dOFV, difference in objective function value between model run including AUC as a covariate and model run excluding AUC as a covariate.

**P* calculated using OFV-based likelihood ratio test (χ^2 with 1 degree of freedom representing the inclusion or exclusion of AUC as a covariate).

Supplementary Figure 1 Raw trajectories and medians of groupings of World Health Organization (WHO) smear grade (low: negative (0) and 1+; high: 3+ and 4+) for rifampin **(A)** and rifapentine **(B)**. A heat map indicating the proportion of observations available at each (nominal) study time (weeks) that lies at the censoring limit (44 days) is included above each raw data plot.

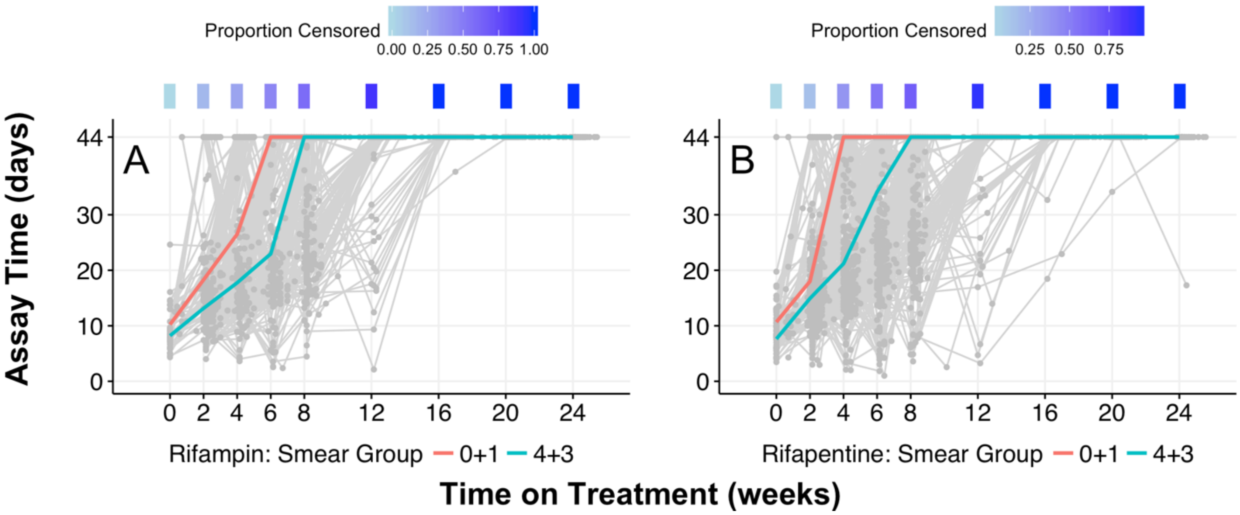

Supplementary Figure 2 Schematic of the effects of changes in population parameter values on the shape of the shifted logistic curve used to define the empirical model for Mycobacteria Growth Inhibitor Tube (MGIT) assay time versus time on treatment. In each plot, the medium-colored curve represents the population-average base model trajectories for rifapentine; the darker curve shows the effect of an increase, and lighter curve shows the effect of a decrease, in the magnitude of the parameter on the shape of the curve and associated decreased model-predicted time-to-culture conversion. In this model, all parameters are constrained to be nonnegative. The dotted portions of the curves define portions above the upper limit of quantification (dashed line), above which observations are censored; complete curves are shown to emphasize the shape of the model. For reference, the equation defining the shifted logistic function from Supplementary Table 1 is repeated here, with parameters color-coded to match the trajectories in the plots.

$$t_{assay} = t_{base} + \frac{t_{max} - t_{base}}{1 + \exp\{-\alpha(t_{treatment} - t_{shift})\}}$$

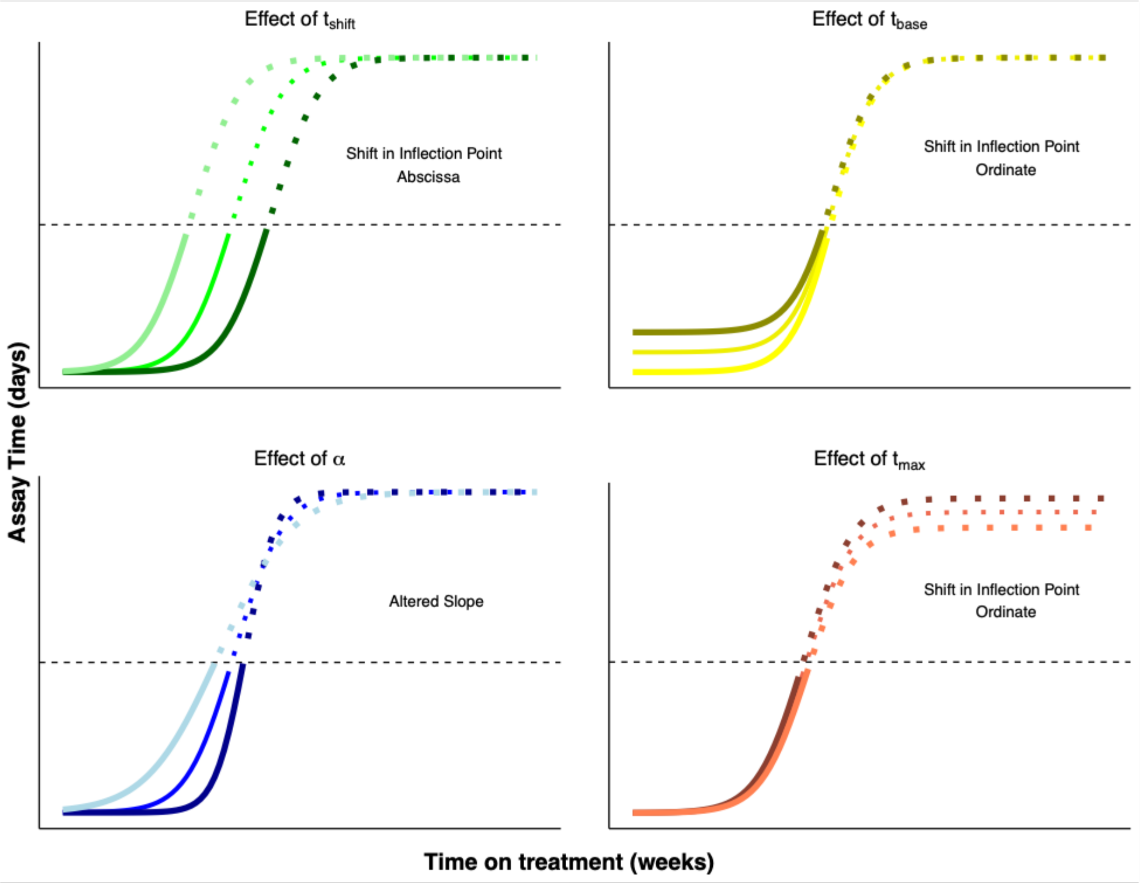

Supplementary Figure 3 Forest plot of Mycobacteria Growth Inhibitor Tube (MGIT) assay model-predicted times to have culture conversion, stratified by selected covariate combinations for rifampin (blue) and rifapentine (red). Simulated 99th percentiles (dots) and 95th-99th percentile ranges (horizontal bars).

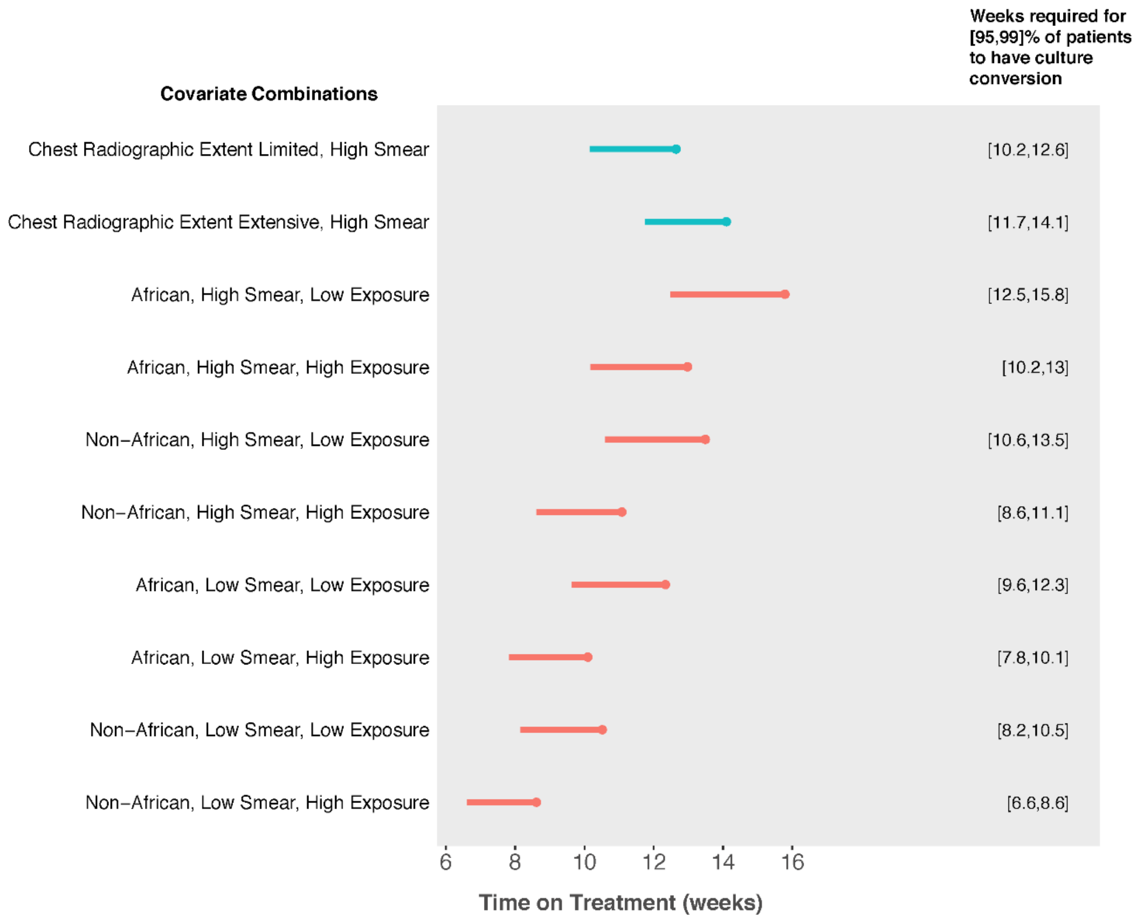

Supplementary Figure 4 Model-predicted time-to-culture conversion (adjusted to next observation) vs actual time-to-culture conversion for rifapentine-treated subjects using the base model plus individual estimates of random effects. Predictions are shown with the data set truncated at 16, 12, 8, and 6 weeks. Gray shaded areas represent individual predictions of 16 weeks or greater.

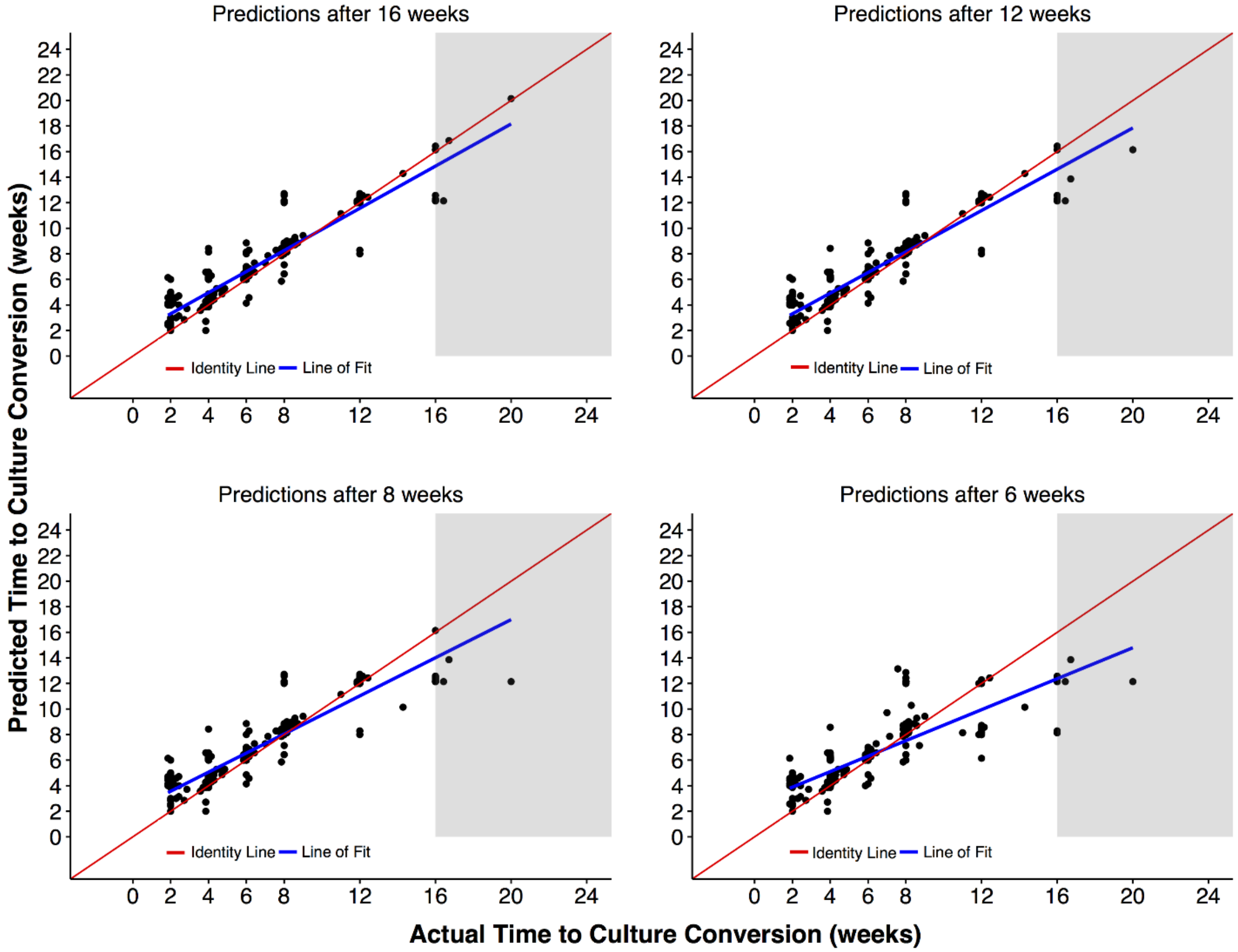

Supplement: Supplemental file 1 — Supplemental tables and figures. Download AAC.01794-20-s0001.pdf, PDF file, 0.8 MB [file aac.01794-20-s0001.pdf]
